# Tumor-Infiltrating Lymphocytes (TILs) as a Biomarker of Abscopal Effect of Cryoablation in Breast Cancer: A Pilot Study

**DOI:** 10.1245/s10434-021-11157-w

**Published:** 2022-01-29

**Authors:** Sonia Y. Khan, Michael W. Melkus, Fahmida Rasha, Maribel Castro, Victoria Chu, Luis Brandi, Hafiz Khan, Harvinder Singh Gill, Kevin Pruitt, Rakhshanda Layeequr Rahman

**Affiliations:** 1 Department of Surgery, School of Medicine, Texas Tech University Health Sciences Center, 3601 4th Street, Lubbock, TX 79430-8312 USA; 2 Department of Immunology and Molecular Microbiology, Texas Tech University Health Sciences Center, Lubbock, TX USA; 3 Department of Pathology, School of Medicine, Texas Tech University Health Sciences Center, Lubbock, TX USA; 4 Department of Public Health, Julia Jones Matthews, Texas Tech University Health Sciences Center, Lubbock, TX USA; 5 Department of Chemical Engineering, Texas Tech University, Lubbock, TX 79409 USA; 6 Breast Center of Excellence, Texas Tech University Health Sciences Center, Lubbock, TX USA

## Abstract

**Background:**

Morphological evaluation of tumor-infiltrating lymphocytes (TILs) in breast cancer is gaining momentum as an immunological biomarker. This experiment evaluates the role of TILs in distant tumors as a measure of abscopal effect from cryoablation of breast cancer.

**Methods:**

BALB/c mice underwent bilateral orthotopic transplant with 4T1-12B (triple-negative) cells. At 2 weeks, left tumors were treated by either resection (standard of care group) or cryoablation (intervention group) followed by resection of the distant right tumors 1 week posttreatment. TIL scores were calculated from hematoxylin and eosin-stained sections and phenotyped for cytotoxic T-lymphocyte (CTL) markers by immunofluorescence. Primarily resected tumors served as baseline (T_baseline_), whereas resected distant right-sided served as the readout for abscopal effect (Abs_Res_ or Abs_Cryo_). Mice were monitored for tumor recurrence and metastasis.

**Results:**

The Abs_Cryo_ had a significant mean (SD) increase in stromal (2.8 [1.1]%; *p* = 0.015) and invasive margin TILs (50 [12]%; *p* = 0.02) compared with T_Baseline_ (1.0 [0]% and 31 [4.9]%, respectively). CTL phenotyping revealed a significant increase in mean (SD) CD8^+^ T cells (15.7 [12.1]; *p* = 0.02) and granzyme B (4.8 [3.6]; *p* = 0.048) for the Abs_Cryo_ compared with T_Baseline_ (5.2 [4.7] and 2.4 [0.9], respectively). Posttreatment, the cryoablation group had no recurrence or metastasis, whereas the resected group showed local recurrence and lung metastasis in 40% of the mice. Postprocedure increase in TIL score of distant tumors was associated with decrease in tumor relapse (*p* = 0.02).

**Conclusions:**

Cryoablation induced a robust tumor-specific TIL response compared with resection, suggesting an abscopal effect leading to the prevention of cancer recurrence and metastasis.

The concept of an immune-mediated response to target metastatic tumor cells located away from the locally treated tumor bed has existed for more than 60 years. While Mole coined the term “abscopal” (“ab” – away from, “scopus” – target) to describe this idea in regards to ionizing radiation,^1^ it is now applied to any form of truly local treatment, such as oncolytic virotherapy,^2^ histotripsy,^3^ and ablative techniques^4,5^ that result in a systemic immune response. Although uncommon, a 2015 study found 46 reported cases of the abscopal effect seen with local radiation therapy alone from 1969 to 2014.^6^ While attempts are still being made to elucidate the biological mechanism behind the abscopal effect, one popular theory regarding the abscopal effect seen with radiation lies with the liberation of tumor-associated antigens (TAAs) upon tumor stress or injury.^7^ A considerable boost in TAAs could stimulate a tumor-specific immune response. These TAAs would then be engulfed by antigen-presenting cells (APCs) and subsequently be presented to CD8^+^ T cells.^8^ Because the basis of the abscopal effect is an increase in immune response, it reflects a systemic link to local response.

Immune cell infiltration of tumors has been shown to provide predictive prognostic value in various tumor types, including lung, colon, ovarian, and breast cancers.^9–12^ In the case of breast cancer, tumor-infiltrating lymphocytes (TILs) have particular value.^12^ Murine and human studies have shown that most leukocyte subtypes predominantly contribute to either a protumor or antitumor microenvironment. For example, increased tumor infiltrating CD8^+^ T cells in breast cancer patients have antitumor activity and are associated with increased patient survival^13^ and response to chemotherapy.^14^ However, some T-cell subtypes, such as Th2 cells, have been associated with a decreased antitumor response.^15^ The effects of tumor-infiltrating B cells are not currently well known.^16^ Despite a lack of knowledge of the immune subpopulation comprising TILs, simple hematoxylin and eosin (H&E)-stained tumor evaluation of the lymphocytic infiltration measured by TIL score can provide predictive and prognostic value in triple-negative breast cancer (TNBC) and human epidermal growth factor receptor 2 (HER2^+^) breast cancer.^12,17–20^ TILs are an important component of the local tumor microenvironment. Because they have prognostic value as demonstrated by correlation with survival,^17^ and predictive value as demonstrated by correlation with pathological complete response to neoadjuvant chemotherapy,^21,22^ we sought to determine whether the TIL score of distant (untreated) tumors has value as a proxy measure of abscopal effect from the cryoablation-induced immune response in the primary tumor.

Cryoablation, unlike traditional resection, which removes the tumor completely, is a method of killing cancer cells via rapid freeze/thaw cycles while leaving the dead tumor cells in vivo. Cryoablation rapidly deep-freezes (≤  − 40 °C) the tumor, thus killing it while preserving potential TAAs, unlike other ablative techniques, such as thermal-heat ablation, which denatures tumor antigens.^23^ Because the ablated tumor remains in the patient, this introduces the potential for augmentation of an immune response to TAAs in vivo.^24^ Kumar et al. presented a case highlighting potential abscopal effect on axillary metastasis after cryoablation of breast cancer.^25^ However, there is no reliable way to measure the abscopal effect in the setting of early-stage disease (without demonstrable metastasis) where cryoablation is most effective.

In this experiment, we aimed to determine whether TILs in the distant tumor could be used as a biomarker for the abscopal effect following primary tumor treatment with cryoablation. We utilized a 2-tumor murine breast cancer model and compared TILs in H&E-stained specimens of unmanipulated distant tumors after treatment of the primary tumors by either cryoablation or resection to measure abscopal effect.

## Methods

### Experimental Design: A Two Mammary Tumor Mouse Model to Evaluate the Abscopal Effect Post-Primary Tumor Treatment

**Fig. 1:**
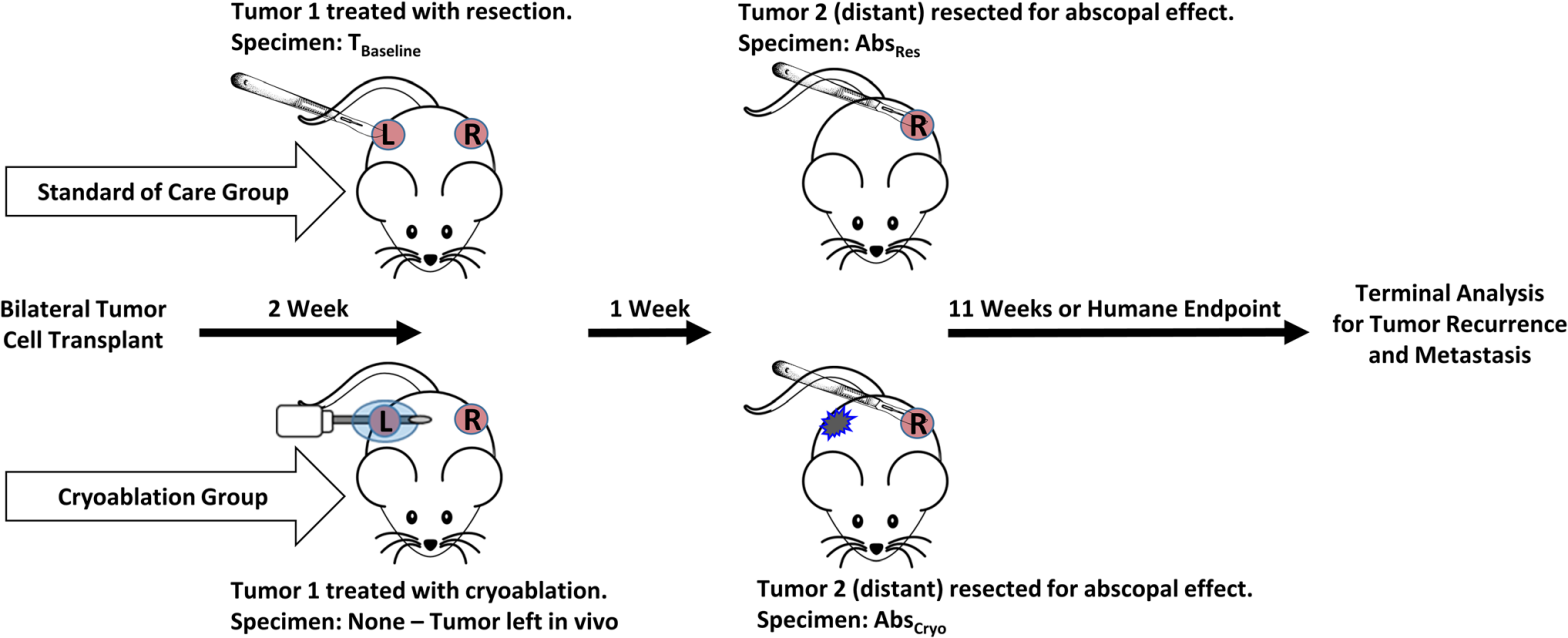
Experimental approach. Mice were transplanted orthotopically with 1 × 10^6^ 4T1-12B cells in the mammary fat pad on each side to establish a 2-tumor breast cancer model to evaluate the abscopal effect post-tumor treatment comparing resection vs. cryoablation. At 2 weeks post-tumor cell transplant, the left tumor was treated either by resection or cryoablation. The resected tumor was scored for TILs and served as the baseline control (T_Baseline_). One week after treatment, the untreated right distant tumor was resected (Abs_Res_ and Abs_Cryo_) for TIL scores to measure the abscopal effect. Mice were then monitored for primary tumor recurrence and metastasis

To address whether TILs in the distant tumor could serve as a biomarker of the abscopal effect resulting from local therapy of breast cancer, we used a 2-tumor mammary cancer mouse model. We utilized one tumor as a target for local therapy (either resection or cryoablation), followed by harvesting of the second, unmanipulated tumor to study the effect of the first treatment. A detailed experimental approach is described in Fig. 1. Local therapy represented two experimental arms: standard of care arm, and resection and intervention arm, i.e., cryoablation. Specimens collected were labeled according to their biological representation as follows:

T_Baseline_ – Treatment naïve tumor representing innate TIL response to tumor antigens.

Abs_Res_ – Distant tumor after local treatment of first tumor with resection representing TIL response affected by systemic effect of resection of first tumor.

Abs_Cryo_ – Distant tumor after local treatment of first tumor with cryoablation representing TIL response affected by systemic effect of cryoablation of first tumor.

### Cell Lines and Animals

The mammary carcinoma 4T1-12B cell line (triple-negative) expresses luciferase for *in vivo* tracking of metastasis was obtained from TUFTS University (kindly provided by Sahagian et al).^26^ Cells were grown in Dulbecco’s Modified Eagle’s medium (DMEM) supplemented with 10% FBS plus 1% penicillin/streptomycin at 5% CO_2_. Female BALB/c mice, aged 8–10 weeks, were purchased from Jackson Labs (Bar Harbor, ME). All work was conducted in accordance with TTUHSC-IACUC policies and approved protocol 17024.

### Tumor Cell Transplant

4T1-12B cells (p22–p24) were passaged at 40–50% confluency 24 hours prior to mouse transplant. Cells were isolated and resuspended at 2 × 10^7^cells/ml in PBS. The mouse model was established by bilateral orthotopic injection with 4T1-12B (1 × 10^6^/side) cells with a 27-gauge needle into the mammary fat pad near nipple 4 and 9 regions.

### Monitoring Tumors

Mice were observed three times per week for overall health and tumor growth. Tumors were monitored and volumes calculated using calipers and the formula V = (W(2) × L)/2. *In vivo* imaging (IVIS Lumina XR (Caliper Life Sciences, PerkinElmer™), mice were administered 100 µl of luciferase substrate (15 mg/ml) intraperitoneally (IP) and imaged after 10 minutes under anesthesia with 2.5% isoflurane. Mice were imaged weekly for tumor growth, metastasis, and tumor recurrence.

### Surgical and Cryoablation Procedures

**Fig. 2:**
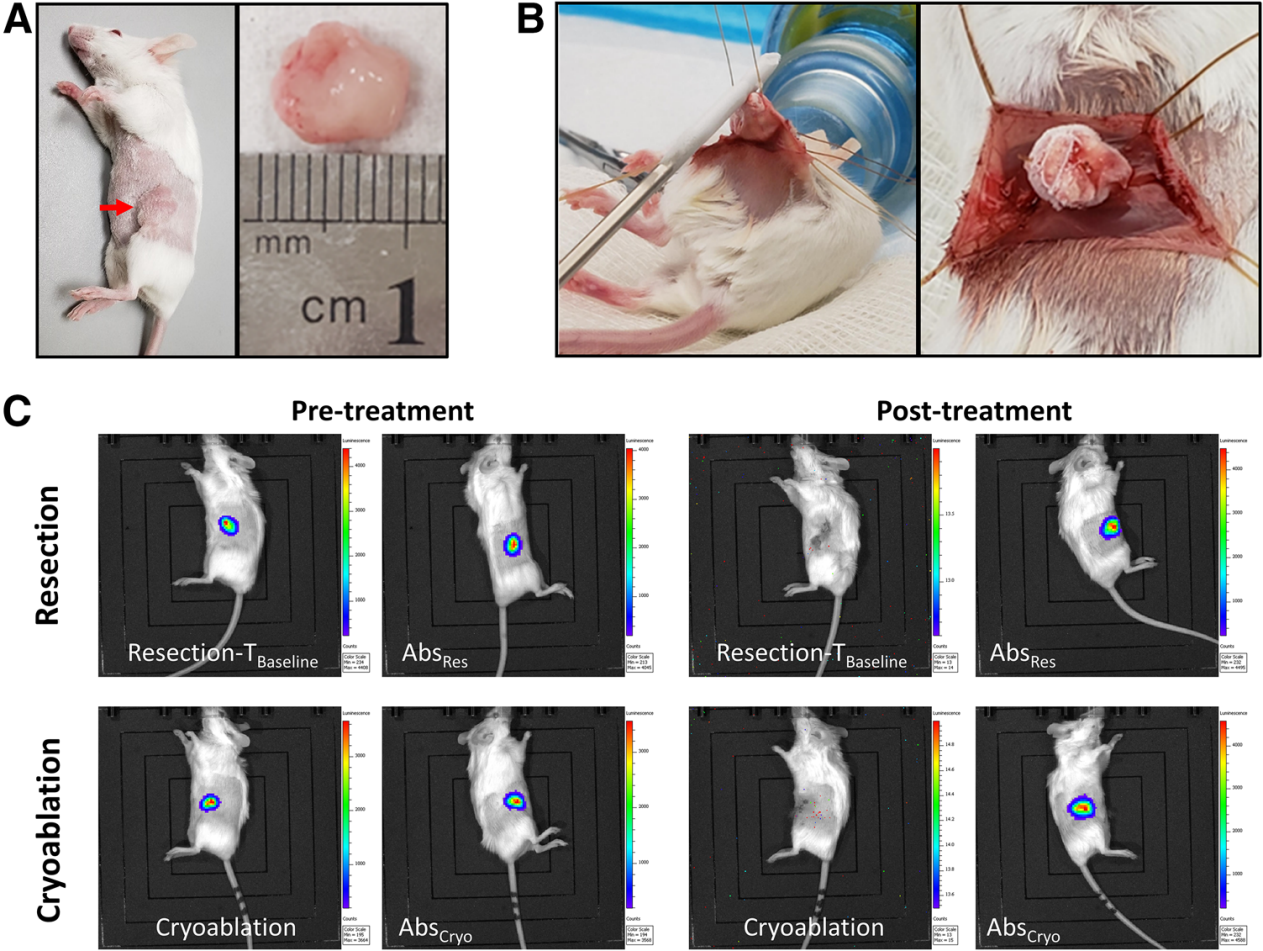
Tumor treatment procedures. **A** Left, tumor growth in mammary fat pad (red arrow); right, resected mammary tumor (serves as baseline control). **B** Left, cryoablation technique freezing the tumor; right, completely frozen tumor – remains in the mouse. **C** IVIS analysis showing tumors before and posttreatment. Both resection and cryoablation treatments of the left tumor showed no luciferase activity 24 hours posttreatment

All procedures were performed under strict aseptic technique. For mice in the standard of care group (*n* = 5), resections were performed with grossly negative surgical margins (Fig. 2A). The wound was then closed using 4-0 Prolene sutures (Ethicon, Somerville, NJ).

For mice in the cryoablation group (*n* = 5), the skin was incised and retracted away from tumor with stay sutures to prevent frostbite. Cryoablation was performed using the liquid nitrogen-based Visica^®^ 2 Treatment System (Sanarus Technologies Inc., Pleasanton, CA) (Fig. 2B). The cryoprobe was placed directly on the tumor mass, and cryoablation was performed at a high rate of freeze with each tumor undergoing a freeze/thaw/freeze cycle. After completion of the cryoablation, the tumor was allowed to thaw and skin was closed over the tumor.

After local therapy (surgical resection or cryoablation), mice were administered 0.05 mg/kg of buprenorphine (Par Pharmaceuticals, Chestnut Ridge, NY) intraperitoneally in 200 µl of saline. Mice were provided MediGel Hazelnut (Clear H_2_O^®^, Portland, ME) supplemented with 0.5 mg/ml of Rimadyl (Zoetis, Parsippany, NJ) for pain management and monitored for 5 days for postsurgical/cryoablation complications.

### Pathology

Standard H&E staining of paraffin-embedded tissue was used for histopathological examination of primary and metastatic tumors. Sections were examined and photographed using a Leica Model DM 2000 LED microscope (Buffalo Grove, IL) equipped with an MC 170 HD camera and Leica Application Suite version 3.4 software.

### TIL Scoring

The evaluation of TILs was based on the guidelines set forth by the International TILs Working Group 2014^17^ and updates from the International Immuno-Oncology Biomarker Working Group on Breast Cancer.^21^ For this study, we analyzed stromal, intratumor, and invasive margin TILs in all tumors to gather the broadest scope of data for correlations between tumor treatment and abscopal effect.

To analyze TILs, an appropriate area was selected that reflected the immune infiltrate present throughout the entire specimen. Once a representative area was found at a low magnification, the magnification was then increased to  × 200–400 to determine the types of inflammatory infiltrate and provide more accurate scoring. TIL scoring was determined by using the area occupied by TILs as the numerator and the local tissue in the field as the denominator. This was repeated with three representative areas. Then, the TIL score was averaged to provide the most accurate score for the specimen.

Stromal TILs are dispersed lymphocytes that are located within the tumor border but do not make direct contact with any tumor cells. Intratumor TILs have intimate cell-to-cell contact with tumor cells with no stromal separation. Invasive margin TILs are similar to stromal; however, they are in the peripheral region of the tumor.

To provide the most accuracy, each of the 3 categories (stromal, intratumor, and invasive margin) of the 15 specimens (5 T_Baseline_, 5 Abs_Res_, and 5 Abs_Cryo_) were independently scored by two trained professionals and then averaged. During analysis, necrotic areas, crush artifacts, healthy tissue, fibrous areas, and focusing on hotspots were avoided.

### Immunofluorescence Staining

TILs also were quantitated for cytotoxic T cells by immunofluorescence staining with biomarkers for CD3ε, CD8α, and granzyme B. Tumor sections were deparaffinized, underwent heat-induced antigen retrieval, and were incubated overnight at 4 °C with rabbit polyclonal antibodies CD3ε (D4V8L; Cell Signaling) at 1:100, CD8α (D4W2Z; Cell Signaling) at 1:100, or granzyme B (EPR22645-206; Abcam Inc) at 1:200 dilution in blocking buffer (PBS, 0.1% Saponin, 5% BSA). Slides were washed with PBS and incubated with an Alexa Fluor 568-conjugated secondary goat anti-rabbit IgG (A11036, Thermo Fisher Scientific) at 1:300 dilution, washed, and mounted with Prolong™ Gold with DAPI (Invitrogen). Image collection and analysis were performed under blinded conditions. Samples were imaged using a confocal microscope Nikon T-1E with a 40x objective and NIS software then analyzed using AdipoGauge software for Windows (Version 2.0).^27^ Antibody fluorescence was normalized using TRITC/DAPI staining for each sample with the lowest reading for each antibody (CD3ε, CD8α, granzyme B) in the baseline group being set at 1 and the other samples calculated as fold change.

### Necropsy

Mice were euthanized using CO_2_ followed by exsanguination and cardiac puncture according to AVMA Guidelines for the Euthanasia of Animals: 2020 Edition. Animals necropsied with the following tissues collected for analysis: mammary tumors, spleens, liver, lungs, femurs, kidneys, heart, and spines. Tumor volume was calculated using calipers measurements. All tissues were visually examined for metastasis and the liver, lungs, spleen, and tumors were analyzed with histopathology by H&E.

### Statistics

All statistical analyses were performed using GraphPad Prism version 9.00 for Windows, GraphPad Software (La Jolla, CA, www.graphpad.com), and SPSS Software version 25 (Armonk, NY, www.ibm.com). A paired *t*-test was used to compare the means of the same group under two separate conditions. An unpaired or independent samples *t*-test was used to compare the means of two unrelated groups. The differences between group scores were considered statistically significant when *p* < 0.05.

## Results

All mice successfully developed bilateral tumors and were subsequently divided into two experimental groups for local treatment: (i) Standard of care - Resection group, and (ii) Cryoablation group. The mean (SD) tumor volume for T_Baseline_, Abs_Res_, and Abs_Cryo_ was 279 (41) mm^3^, 276 (122) mm^3^, and 224 (56) mm^3^, respectively. There was no significant difference for tumor volume or weight between any of the three tumor groups. Cryoablated tumors remained in vivo and showed complete destruction by *in vivo* imaging (Fig. 2). For the cryoablation group, the average freeze/thaw/freeze cycles were 84/180/81 seconds. At 24-hour posttreatment assessment of the two local treatment procedures, no mice showed luciferase activity at the treatment site, indicating complete tumor ablation or tumor resection; however, the nontreated distant tumors still had a high fluorescent measurement (Fig. 2C).

**Fig. 3:**
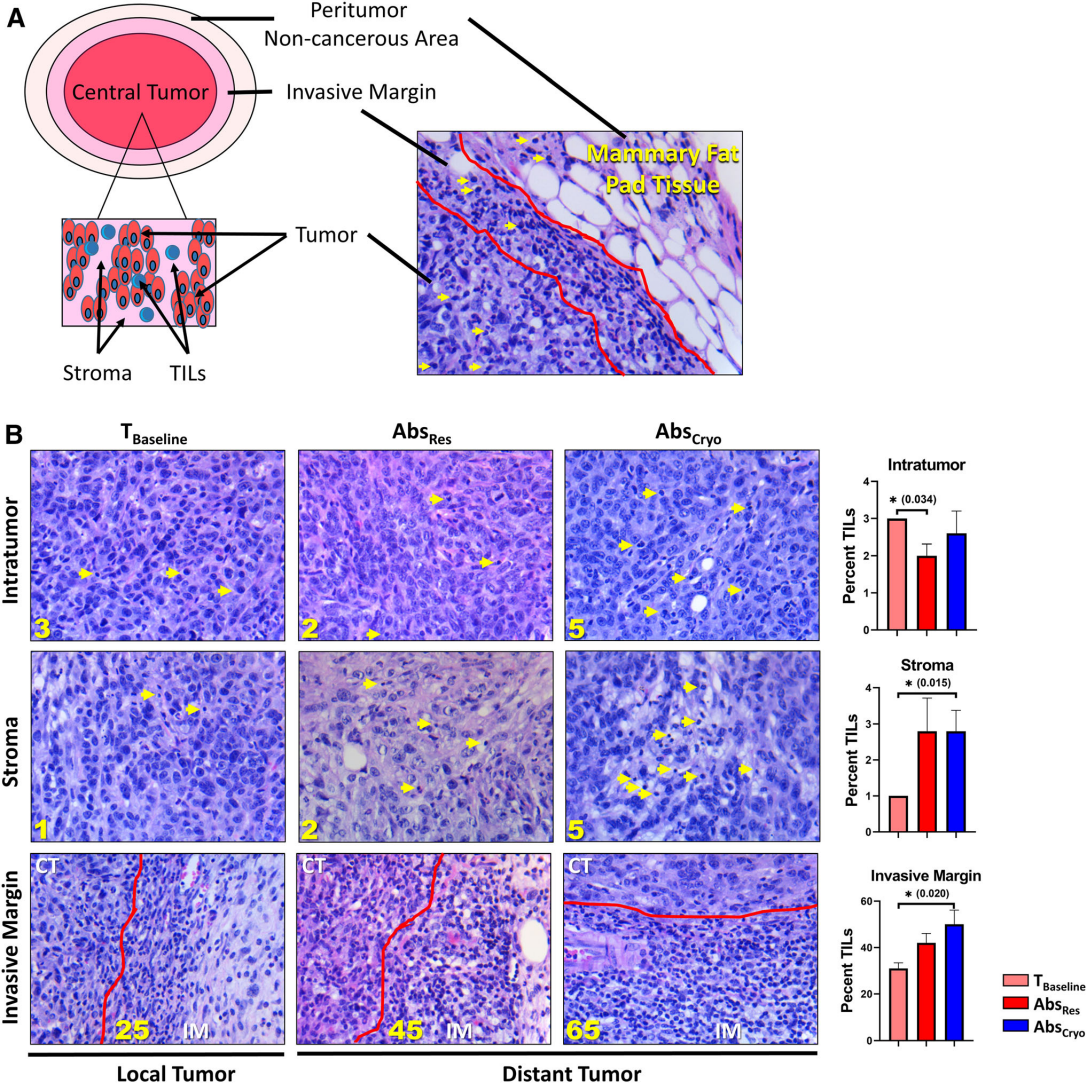
Tumor-infiltrating lymphocyte (TIL) scores. **A** Left, tumor schematic showing regions of lymphocytes in the tumor for TIL scoring; Right, H&E histopathology demonstrating TILs in each region of the tumor used to score TILs. **B** Representative TIL analysis for the resected baseline control (T_Baseline_) and the distant tumor (Abs) from each treatment group showing a low, medium, and high TIL score. The tumors were analyzed for intratumor, stroma, and invasive margin TILs. Graphs on the far right are group mean with SD. There was a significant increase in stromal and invasive margin TILs for the Abs_Cryo_ group compared with baseline with significance considered *p < 0.05

One week after local treatment, the distant tumor was resected and labeled according to schema based on local therapy of primary tumors (Abs_Res_ or Abs_Cryo_). All tumors were analyzed for TILs (Fig. 3). T_Baseline_ were remarkably consistent for overall TIL percentages. There was no correlation between TIL scores to tumor volume with all resected tumors having a stromal TIL score of 1% (Fig. 3B; Table 1). Both Abs_Res_ and Abs_Cryo_ had an overall increase in stromal and invasive margin TILs compared with T_Baseline_. There was a significant increase in the mean (SD) stromal (2.8 [1.1]%; *p* = 0.015) and invasive margin (50 [12.2]%; *p* = 0.02) TILs for the Abs_Cryo_ group compared with T_Baseline_ of 31 (4.9%) (Fig. 3; Table 1). The Abs_Res_ did not show significant increase from T_Baseline_.

**Table 1:** Tumor characteristics and TIL scores

TIL analysis						
Mouse	Volume (mm^3^)	Intratumor (%)	Stroma (%)	Invasive margin (%)	Tumor recurrence	Metastasis
*Baseline – resection treatment control group (T*_*Baseline*_*)*						
M1	286	3	1	40	N/A	
M2	327	3	1	30		
M3	317	3	1	30		
M4	223	3	1	25		
M5	240	3	1	30		
Mean ± SD	279 ± 41	3 ± 0.0	1 ± 0.0	31 ± 4.9		
*Abscopal tumor - resection group (Abs*_*Res*_*)*						
M1	502	2	5	40	No	No
M2	185	2	5	40	No	No
M3	218	1	1	55	Abscopal site	Lung
M4	303	2	2	45	No	No
M5	172	3	1	30	Treatment site	Lung
Mean ± SD	276 ± 122	2 ± 0.6	2.8 ± 1.8	42 ± 8.1	2/5 (40%)	2/5 (40%)
*Abscopal tumor- cryoablation group (Abs*_*Cryo*_*)*						
M6	303	5	5	65	No tumor recurrence No metastasis	
M7	248	2	2	50		
M8	218	2	3	60		
M9	223	2	2	30		
M10	130	2	2	45		
Mean ± SD	224 ± 56	2.6 ± 1.2	2.8 ± 1.1	50 ± 12.2	0/5 (0%)	0/5 (0%)

**Fig. 4:**
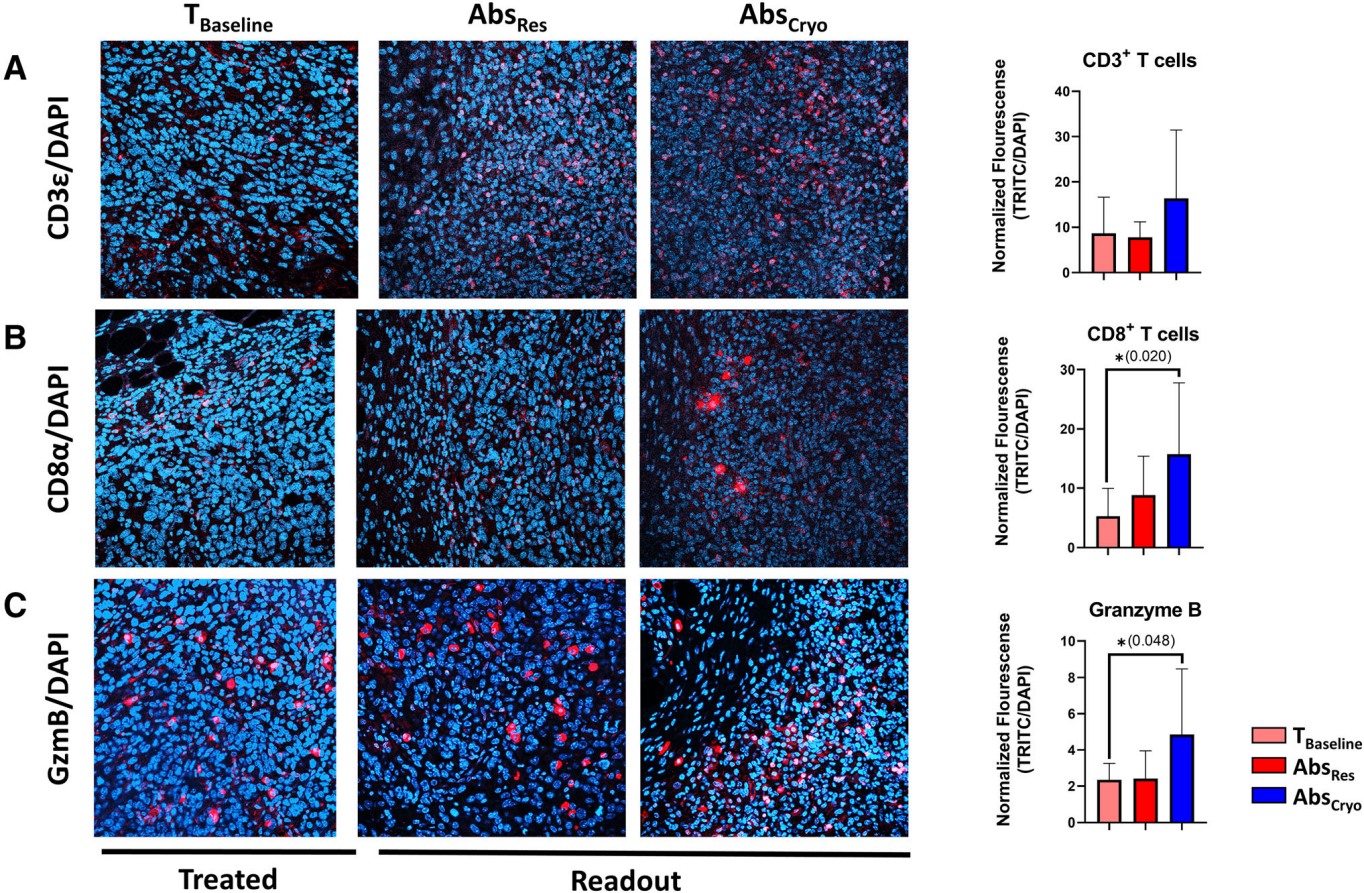
TIL phenotyping for cytotoxic T lymphocytes. Immunofluorescent staining for CD3^+^ T cells, CD8^+^ T cells, and granzyme B—a cytotoxic T-cell marker. Data shown are for T_Baseline_, Abs_Res_, and Abs_Cryo_ tumors stained with **A** anti-CD3ε (red), **B** anti-CD8α (red), **C** anti-granzyme B (red) and DAPI (blue). Graphs on the far right are group mean with SD for normalized fluorescence for each antibody marker. The cryoablation group shows increased CD3ε staining and a significant increase of CD8α and granzyme B staining compared with baseline control tumors, with significance considered *p < 0.05

TILs were further assessed for changes in CTL markers in the Abs_Res_ and Abs_Cryo_ compared with T_Baseline_ by immunofluorescent staining for CD3ε (T cells), CD8α (CTLs), and granzyme B (activated CTLs) (Fig. 4). The Abs_Cryo_ tumors had an overall increase in CD3ε normalized fluorescence staining compared with both the T_Baseline_ and Abs_Res_ tumors. There was a slight increase in CD8α staining in the Abs_Res_ group compared with T_Baseline_, but virtually no change in CD3ε or granzyme B staining. It is noteworthy that in the Abs_Cryo_ tumors, there was a significant increase in mean (SD) normalized fluorescence for CD8α (15.7 [12.1]; *p* = 0.020] and the CD8 CTL activation marker granzyme B (4.8 [3.6]; *p* = 0.048) compared with T_Baseline_ (5.2 [4.7] and 2.4 [0.09], respectively), indicating increased activated CTLs in the tumor (Fig. 4).

**Fig. 5:**
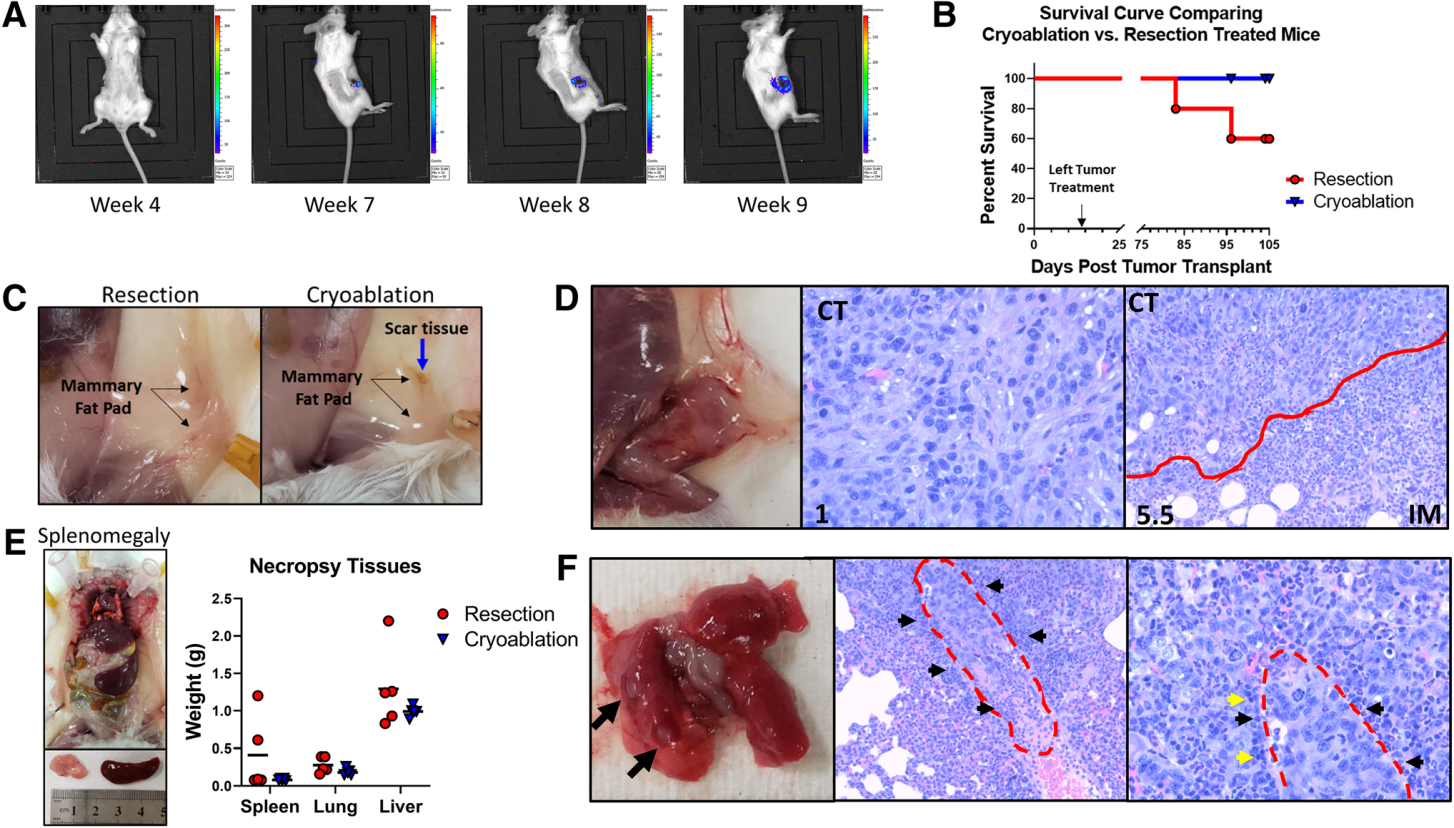
Tumor recurrence and metastasis. **A** IVIS analysis showing primary tumor recurrence post-cryoablation. **B** Survival curves for resected vs. cryoablated mice. **C** Necropsy: Left, comparison of tumor site post-tumor treatment, the cryoablated tumor was completely reabsorbed leaving only small residual scar tissue. **D** Left, gross tissue showing primary tumor recurrence and right, H&E analysis for TILs. **E** Gross tissue showing splenomegaly and graph of tissue weights for spleen, lung, and liver. **F** Left, lung metastasis nodules. Black arrows indicate tumors. Right, histopathology for lung metastasis. Red dash outlines metastatic tumor zone, black arrows indicate neutrophils and yellow arrows indicate TILs

An evaluation comparing the standard of care group to the cryoablation group showed that cryoablation was superior to resection in preventing tumor recurrence (Fig. 5). At 4 weeks post-cryoablation, all mice were tumor-free by palpation and IVIS analysis. At 6 weeks post-cryoablation, two mice in the resection group had pin-point tumors at the resection sites by palpation, which for one of the mice was easily detected by IVIS by 7 weeks (Fig. 5A). Two of five (40%) mice in the resection group versus none of the five mice in the cryoablation group developed local and systemic failure of disease. Both tumor-positive mice had to be sacrificed due to humane endpoints, whereas the cryoablation group all appeared healthy and showed no signs of disease at sacrifice time points (Fig. 5B). Moreover, the cryoablation procedure showed minimal scar tissue with healthy mammary tissue at time of sacrifice (Fig. 5C). Of the mice in the resection group that developed tumor recurrence, one was at the initial resection-treated tumor site and the other was at the excised Abs_Res_ site (Fig. 5D; Table 1). Neither tumor had substantial number of TILs with 1% central tumor and less than 6% invasive margin TILs. Of note, two of the five mice in the resection group that did not mount a central tumor TIL response were the same mice that failed long-term control of tumor growth. Proportion test showed significant association between increase in TIL% and prevention of tumor recurrence (*p* = 0.02). Overall, TIL scores for the recurrent primary tumors was much lower than any of the previous tumor TIL scores analysis. The stroma TILs remained at 1%, whereas the intratumor TILs decreased to 0% and the invasive margin TILs decreased to 3.5% and 5.5%. Mice that developed tumor recurrence had increased spleen and liver weights (Fig. 5E) compared with the other mice with both mice developing splenomegaly (Fig. 5E) most likely due to tumor-induced granulopoiesis. Additionally, mice that had tumor recurrence exhibited metastasis to the lung (Fig. 5F) and suspicious regions in the liver (data not shown). These results indicate that local cryoablation therapy generated a strong systematic antitumor immune response and long-term antitumor immunity that protected against tumor recurrence and metastases.

## Discussion

Among the hallmarks of cancer is a competition between the tumor attempting to escape immune surveillance and antitumor immunity versus the immune system recognizing and eliminating the tumor.^28^ This phenomenon is being extensively investigated in the local and systemic environment created by the tumor and attempted therapies. Whereas local antitumor immunity is studied within the tumor microenvironment, the systemic response is a subject of investigation in terms of abscopal effect. The tumor microenvironment, comprised of immune cells, signaling molecules, extracellular matrix, adjacent blood vessels, and fibroblasts, is the battleground and has the potential to provide an advantage to one side, depending on its composition. Immune cells, in particular, play an essential role in this battle by providing the factors that comprise the tumor microenvironment—both adding to the antitumor defenses and blocking them. Even within lymphocytes, there are subtypes on either side. For example, tumor infiltration by CD8^+^ effector T cells is associated with significant antitumoral response and longer survival in breast carcinoma.^13^ Conversely, T_Reg_ cells, which normally suppress self-reactive T cells to fight autoimmune diseases, are believed to contribute to tumor tolerance by suppressing other immune cells.^13^ However, the overall percentage of lymphocytes in the tumor bed observed without subtyping are still predictive of better prognosis in TNBC and HER2^+^ breast cancer.^12,18–20^

Breast cancer TIL score predicts the effectiveness of breast cancer treatment and prognosis.^12,18–20^ Breast cancer patients with a higher TIL score on diagnostic biopsy specimens appear to do better with adjuvant and neoadjuvant systemic therapy and result in less tumor metastasis.^12,18–20^ The robust evidence supporting TIL scoring as an important prognostic marker led to the formation of the International Immuno-Oncology Biomarker Working Group on Breast Cancer that recommends regular TIL reporting on breast carcinomas following the guidelines provided in their 2017 report.^21^ However, TIL scores have mainly been reported within the context of primary tumor. To the best of our knowledge, no studies have looked at the role of TIL scores in distant tumors as a potential biomarker of abscopal effect resulting from local therapy of primary tumor.

It is well established the systemic immune response plays the predominant role in executing the abscopal effect.^29^ Previous studies have shown that primary tumors treated by ablation exerts a direct cytotoxic effect on the tumor cells and reprograms the tumor microenvironment.^30^ Tumor ablation initiates immunogenic cell death (ICD) releasing damage associated molecular pattern (DAMPs) molecules, cytokines, and chemokines, which recruit immune cells into the tumor and induces potent antitumor immune responses with increases in dendritic cell antigen presentation and activation of CD8^+^ T cells.^31^ These activated CD8^+^ CTLs then target the primary treated tumor and also can enter into the periphery as the prime driver of the abscopal effect targeting distant tumors and metastasis.^32–34^ Therefore, methodology to measure abscopal TILs theoretically could potentially serve as an innovative tool for monitoring and predicting cancer ablation treatment efficacy and long-term survival.

Cryoablation of breast cancer provides a viable alternative to traditional resection and thermal ablation where the tumor undergoes a series of freeze/thaw cycles but remains within the patient.^35^ As the “killed” tumor and surrounding tissue architecture is preserved, there remains the potential for augmentation of an immune response to the preserved TAAs (potentially measurable by TIL scoring),^24^ leading to an abscopal effect. This aligns with our findings of increased stromal and invasive margin TILs in the unmanipulated distant tumors 1 week after local therapy of primary tumor, with a significant increase for stromal and invasive margin TILs in the cryoablation group. Moreover, we demonstrated that the TIL score of distant tumor correlates with systemic tumor burden and survival, albeit in a small sample. Thus, the distant, unmanipulated tumors represent a target to study abscopal effect resulting from the treatment of the first treated tumor using TIL score as a proxy measure for systemic disease control. Interestingly, we also found a lesser increase in TILs after resection of the first treated tumor, albeit not statistically significant. This modest effect could have resulted from the natural inflammatory response secondary to local trauma of handling and resection. Notably, 40% (2/5) of the mice in the resected group developed both tumor recurrence and metastasis to the lung. The same mice were the only ones in the entire experiment (the resected and cryoablated groups combined) with no increase in stromal TILs compared with the baseline control tumors. This suggests that cryoablation provided a more robust antitumor response both locally and systemically, describing an abscopal effect. More importantly, because the relapse was associated with lack of TIL response, TIL counts can be used as a proxy measure for abscopal effect.

While local resection, which is the mainstay for local control in the present time, removes the primary tumor and provides a minimal local inflammatory response, micrometastasis may still exist. More importantly, by removing the TAAs, resection denies an opportunity for the immune system to mount a robust response needed to overcome the potential for targeting metastasis. Cryoablation is clinically used for the treatment of fibroadenomas and low-risk cancers,^35–39^ mainly because of convincing evidence supporting complete destruction of tumors up to 15 mm in size. However, the potential of augmented immune response by virtue of leaving TAAs in vivo, is not very well-leveraged in the present day practice. This raises the concept of expanding cryoablation to high-risk tumors (TNBC and HER2^+^) that are more immunogenic^40^ but have a poor prognosis to conventional treatments. Cryoablation may provide the immunologic boost needed for improved prognosis in these already immunogenic tumors.

Additionally, cryoablation could be combined with immune modulators to create a more robust immune response. Immune modulators target and inhibit checkpoint molecules like cytotoxic T lymphocyte antigen-4 (CTLA-4) and programmed death-1/ligand-1 (PD-1/PD-L1). A 2016 pilot study found that the combination of ipilimumab, an immune modulator, with cryoablation in women with invasive breast cancer of any HER2^+^ and hormone receptor status led to peripheral elevations in Th1-type cytokines, activated and proliferating CD4^+^ and CD8^+^ T cells, and post-treatment proliferative T-effector cells relative to T-regulatory cells within the tumor. Study with a larger sample size focused on already immunogenic tumors could be performed to determine the potential benefit.^41^ As these studies on local therapies alone or in combination move forward in the clinical realm, it will be important to identify relevant and pragmatic biomarkers that could be utilized as outcome measures (indicative of impact on survival) to foster cost-effective clinical trials. This translational work is ideally suited for development of animal models that endorse biomarkers that could be successfully translated into clinical research and practice.

Animal models for translational research provide insight into cancers and therapeutic responses that cannot be performed through clinical research studies. Although animal models have successfully helped to define mechanisms of cancer initiation, development, and treatment, there are clearly differences between rodent models and human cancer development. For example, breast cancer in humans tends to spread lymphatically to lymph nodes, then metastatically to the bones, brain, and adrenal gland, and then the liver and lung, whereas in the mouse, metastasis is almost exclusively through the hematogenous route to the lung and liver.^42^ Therefore, when utilizing animal models for cancer research, these differences should be considered for both analysis and interpretation of the results. For our study, we focused our metastasis analysis on the lung and liver; however, the immune system in both humans and mice is systemic and functions similarly. Therefore, correlations for the abscopal effect in the mouse can be extrapolated to humans. We found that metastasis was only observed in the mice that had tumor recurrence. It is possible that local tumor recurrence is a marker of systemic disease rather than the cause of metastasis. While immune cells were present at the site of metastases, they were primarily comprised of neutrophils, while lymphocytes, although present, were sparse (Fig. 5F). This also suggests that the surgical resection did not induce as strong of an antitumor CTL response in these mice to control tumor recurrence and metastasis. Most importantly, these outcomes were predictable in our model via TIL scoring of the abscopal readout tumors. Intuitively, the difference in TIL score from baseline to postablation in humans could potentially predict the desired abscopal effect.

Most previous breast cancer mouse models use a single tumor model, perform cryoablation or resection, and analyze the results for the immune response.^33,34,43^ Sabel et al. demonstrated that cryoablation of MT-901 tumors resulted in a tumor-specific immune response in the tumor draining lymph nodes, which correlated with rejections of tumors upon rechallenge.^33^ In our approach, we designed an experiment using two tumors to evaluate TILs in the distant tumor as an immunological readout to measure the abscopal effect. We found that changes in TIL scores in the abscopal-tumor appear to reflect whether mice developed recurrence and emphasize the importance of TILs for predicting long-term outcomes. Although a larger study is needed to confirm these results, this preliminary study reflects the utility of using TILs for measurement of abscopal effect.

Overall, we found that both resection and cryoablation induce an antitumor response with cryoablation being superior. It is likely that tumor resection results in both detrimental and beneficial effects on the systemic immune system, which might explain why 40% of the mice in the resection group failed to control disease. Surgery can trigger healing programs that lead to induce immunosuppressive states primarily driven by myeloid subsets, which can promote protumorigenic niches and suppress T-cell responses. Conversely, the benefit of resection with sufficient recovery time is reduced primary tumor burden allowing for restored systemic adaptive immune responses.^44^ We observed increased TILs in the distant tumors with a significant increase in stromal and invasive margin TILs for the cryoablation group. Furthermore, TIL phenotyping showed that the Abs_Cryo_ tumor TILs represent an increase in CTL markers by immunofluorescent staining. CD8^+^ T cells only express granzyme B following stimulation and differentiation into cytotoxic T cells.^45^ Remarkably, cryoablation induced a stronger abscopal CTL response with a significantly higher increase in both CD8α and granzyme B staining when comparing the two treatment procedures to baseline tumors. We cannot attribute the granzyme B specifically to only CD8^+^ T cells, because activated CD4^+^ CTLs and NK cells also express granzyme B. However, the data show an increase in immune cytolytic activity in the Abs_Cryo_ tumors. Interestingly, only mice that did not have an increase in stromal TILs posttreatment in the resection group had recurrence and metastasis, suggesting that induced TIL response is important in predicting overall disease control. The TIL score and CTL analysis in combination with the lack of tumors posttreatment showed that cryoablation resulted in an overall greater abscopal effect in preventing tumor recurrence and metastasis. Because the tumor microenvironment influences the TIL response, it will be important to further characterize the TILs for both antitumor and suppressive phenotypes and quantitate the ratios of activated CTLs (CD8^+^ICOS^++^) versus T_Regs_ (CD4^+^FOXP3^+^) posttreatment. These ratios could potentially be used as an additional TIL scoring method for predicted treatment efficacy for cryoablation or novel therapies. Future experiments will focus on using RNA-seq analysis to detect “immune gene signature” differences between resection and cryoablation using gene expression levels of immune-related genes to describe the composition and functional status of the immune cell infiltrates, which could be translated for clinical applications.

Questions remain about why some individuals immunologically respond to treatment better than others and how to enhance the response in poor responders. These could be helped by studying the baseline TIL responses mounted by individuals and the ability of local therapies to enhance this response.

## Conclusions

Primary local treatment of triple-negative breast cancer by both resection and cryoablation increases systemic immune response, albeit the response is superior with cryoablation. Unmanipulated tumor TIL scoring provides a viable biomarker for studying the abscopal effect of local therapies.
